# *MLN51 *and GM-CSF involvement in the proliferation of fibroblast-like synoviocytes in the pathogenesis of rheumatoid arthritis

**DOI:** 10.1186/ar2079

**Published:** 2006-11-14

**Authors:** Jinah Jang, Dae-Seog Lim, Young-Eun Choi, Yong Jeong, Seung-Ah Yoo, Wan-Uk Kim, Yong-Soo Bae

**Affiliations:** 1 Department of Biological Science, Sungkyunkwan University, 300 Cheoncheon-dong, Suwon, Gyeonggi 440-746, Korea; 2 Division of DC Immunotherapy, CreaGene Research Institute, Aramson Plaza, 164-7 Poi-dong, Kangnam-gu, Seoul 135-960, Korea; 3 Division of Rheumatology, Department of Internal Medicine, School of Medicine, Catholic University of Korea, St Vincent Hospital, 93 Chi-dong, Suwon, Gyeonggi 442-723, Korea

## Abstract

Rheumatoid arthritis (RA) is an inflammatory autoimmune disease of unclear etiology. This study was conducted to identify critical factors involved in the synovial hyperplasia in RA pathology. We applied cDNA microarray analysis to profile the gene expressions of RA fibroblast-like synoviocytes (FLSs) from patients with RA. We found that the *MLN51 *(metastatic lymph node 51) gene, identified in breast cancer, is remarkably upregulated in the hyperactive RA FLSs. However, growth-retarded RA FLSs passaged *in vitro *expressed small quantities of *MLN51*. *MLN51 *expression was significantly enhanced in the FLSs when the growth-retarded FLSs were treated with granulocyte – macrophage colony-stimulating factor (GM-CSF) or synovial fluid (SF). Anti-GM-CSF neutralizing antibody blocked the *MLN51 *expression even though the FLSs were cultured in the presence of SF. In contrast, GM-CSF in SFs existed at a significant level in the patients with RA (*n *= 6), in comparison with the other inflammatory cytokines, IL-1β and TNF-α. Most RA FLSs at passage 10 or more recovered from their growth retardation when cultured in the presence of SF. The SF-mediated growth recovery was markedly impaired by anti-GM-CSF antibody. Growth-retarded RA FLSs recovered their proliferative capacity after treatment with GM-CSF in a dose-dependent manner. However, *MLN51 *knock-down by siRNA completely blocked the GM-CSF/SF-mediated proliferation of RA FLSs. Taken together, our results imply that *MLN51*, induced by GM-CSF, is important in the proliferation of RA FLSs in the pathogenesis of RA.

## Introduction

Synovial tissue from healthy individuals consists of a single layer of synovial cells without infiltration of inflammatory cells. In rheumatoid synovial tissue, lymphocytes and macrophages are recruited and activated, and these activated macrophages release high concentrations of inflammatory cytokines. In response to these cytokines, synovial fibroblasts proliferate vigorously and form villous hyperplastic synovial tissues. These fibroblasts secrete inflammatory mediators, which further attract inflammatory cells and stimulate the growth of the synovial fibroblasts and vascular endothelial cells [1]. These activated macrophages and fibroblasts produce tissue-degrading proteinases [2]. Thus, invasive hyperplastic synovial tissue, termed pannus, is directly responsible for the structural and functional damage to the affected joints. Therapeutic intervention against rheumatoid arthritis (RA) could aim at any one of the aforementioned steps, but the driving mechanisms underlying this process are largely unknown. Impaired regulation of apoptosis has been associated with RA [3-5]; however, apoptosis of synovial cells has been identified in rheumatoid synovium [6,7], which suggests that synovial tissue hyperplasia may be a result of cell proliferation rather than apoptotic cell death [8-10].

This study was initiated to address the molecular characterization of fibroblast-like synoviocyte (FLS) hyperproliferation in RA pathogenesis. We used cDNA microarray technology to identify genes related to the proliferation of RA FLSs. We found that the expression of the *MLN51 *(metastatic lymph node 51) gene was markedly enhanced in RA FLSs when cultured in the presence of the RA synovial fluid (SF). *MLN51 *was first identified in breast cancer cells, and the same investigators subsequently reported that *MLN51 *associates with exon junction complexes in the cell nucleus and remains stably associated with mRNA in the cytoplasm [11,12]. Recently, the interactions of *MLN51 *with other exon junction complex components, a clamping mechanism on mRNAs, and some additional biological functions of *MLN51 *in the exon junction complex core have been identified and addressed [13-15].

Our series of experimental results have demonstrated that *MLN51 *is important in the hyperproliferation of RA FLSs in the presence of granulocyte – macrophage colony-stimulating factor (GM-CSF) in SF. These results strongly suggest that the *MLN51 *gene would be an ideal target for the development of new RA therapeutics.

## Materials and methods

### Isolation and establishment of RA FLSs from patients with RA

FLS cells (designated RA s-2, 2–6, 2–14, 2–18, 2–36 and 2–38) were prepared from synovectomized tissue of six patients with RA undergoing joint replacement surgery at the Kangnam St Mary Hospital, Catholic University of Korea, Seoul, Korea. Institutional Board Approval (IRB) and informed patient consent were obtained for each enrolled participant. The mean age of the patients was 43.7 years and their disease duration was greater than 24 months. The patients had visible joint erosions by radiography of the hand, and all satisfied the diagnostic criteria of the American College of Rheumatology (formerly the American Rheumatism Association) for the classification of RA [16]. RA FLSs 2–14, 2–18, 2–36 and 2–38 among the above FLSs could be subjected to Western blot analysis because their sample amounts were sufficient. RA FLSs were prepared as described previously [17-19]. In brief, synovial tissues were minced into pieces 2 to 3 mm in size and treated for 4 hours with 4 mg/ml type 1 collagenase (Worthington Biochemicals, Freehold, NJ, USA) in DMEM at 37°C in 5% CO_2_. Dissociated cells were centrifuged at 500 *g *for 10 minutes and were resuspended in DMEM supplemented with 10% FCS, 2 mM L-glutamine, 100 U/ml penicillin and 100 μg/ml streptomycin. Suspended cells were plated in 75 cm^2 ^culture flasks and cultured at 37°C in 5% CO_2_. Medium was replaced every 3 days, and once the primary culture had reached confluence, cells were split weekly. Cells at passages 5 to 8 were morphologically homogenous and had the appearance of FLSs with typical bipolar configuration under inverse microscopy (less than 2.5% CD14^+^, less than 1% CD3^+ ^and less than 1% CD19^+ ^in flow cytometry analysis) [17]. Osteoarthritis (OA) FLSs (designated OA 2–43, 2–46 and 2–47) were used as controls and were prepared from the synovial tissues of three confirmed and enrolled patients with OA. Synovial fluid samples were obtained from the knee joints of different six patients with active RA.

### Generation of mouse bone marrow-derived dendritic cells

Immature bone marrow-derived dendritic cells (BmDCs) were generated from bone marrow precursor cells of DBA/1J mice (obtained from the Jackson Laboratory, Bar Harbor, ME, USA) as described previously [20]. In brief, bone marrow cells were harvested from the femurs and tibias of mice and plated in RPMI-1640 medium supplemented with 10% FBS, 50 μM 2-mercaptoethanol, and high-dose (200 U/ml) murine GM-CSF (Endogen, Inc., Cambridge, MA, USA). The medium was changed every other day. Seven days later, non-adherent cells (immature DCs) were harvested by gentle washing with warm PBS. For DC maturation, cells were stimulated for 24 hours with TNF-α (500 U/ml; Endogen) or with lipopolysaccharide (*E. coli*, 0127:B8; 1 μg/ml; Sigma-Aldrich, St Louis, MO, USA) together with anti-CD40 (clone 3/23 or HM40, 5 μg/ml; BD Pharmingen, San Jose, CA, USA). The purity and maturation status of DCs were analyzed by a flow cytometer (FACSCalibur; BD Biosciences, San Jose, CA, USA) with the use of fluorescein isothiocyanate-conjugated CD44, CD80, CD86, CD205 and MHC II mAbs or phycoerythrin-conjugated CD11c, CD40 and ICOSL mAbs (BD Pharmingen, San Diego, CA, USA). Data were analyzed with Cell Quest Software.

### DC cell line

BC-1 cells, from the DC cell line generated from BALB/c mouse spleen [21,22], were kindly provided by Dr Onoe (Institute for Genetic Medicine, Hokkaido University, Sapporo, Japan). BC-1 cells were cultured and expanded in Iscove's modified Dulbecco's medium containing 10% FCS, 30% NIH/3T3 culture supernatant, and 10 ng/ml mouse recombinant GM-CSF. Cultured cells exhibit an immature DC phenotype.

### cDNA microarray analysis of rheumatoid arthritis fibroblast-like synoviocytes

Two types of immunologic cDNA microarray chip, namely HI380 and MI380 (Creagene Inc., Seoul, Korea) described previously [23], were used in this study (MI380 microarray data were not shown in the present report. Total RNA was extracted with Trizol reagent (Invitrogen, Carlsbad, CA, USA) and purified by using the RNeasy total RNA isolation kit (Qiagen, Valencia, CA, USA) in accordance with the manufacturer's instructions. The gene expression profile of human RAFLSs and mouse BmDCs were analyzed with the HI380 and MI380 microarray chips, consisting of 384 human and mouse cDNA clones, respectively. Total RNA (20 μg) was reverse-transcribed in the presence of Cy-3-conjugated or Cy-5-conjugated dUTP (Amersham Pharmacia Biotech, Piscataway, NJ, USA), using SuperScript II and oligo(dT)_18 _primer (Invitrogen) in a reaction volume of 20 μl in accordance with the method suggested by the manufacturer. After the labeling reaction for 1 hour at 42°C, unincorporated florescent nucleotides were cleaned up with a Microcon YM-30 column (Millipore, Bedford, MA, USA). The Cy-3-labeled and Cy-5-labeled cDNA probes were mixed together and hybridized to a microarray slide. After incubation overnight at 65°C, the slide was washed twice with 2 × SSC containing 0.1% SDS for 5 minutes at 42°C, once with 0.1 × SSC containing 0.1% SDS for 10 minutes at room temperature, and finally with 0.1 × SSC for 1 minute at room temperature. Slides were dried by centrifugation at 650 r.p.m. for 5 minutes. Hybridization images on the slide were scanned with a Scanarray lite (Packard Bioscience, Boston, MA, USA) and analyzed with GenePix Pro3.0 software (Axon Instruments, Union City, CA, USA). Three separate and independent experiments were performed and the ratio of Cy-3 and Cy-5 signal intensities was calculated for each spot. These ratios were log_2_-transformed and normalized by subtracting the average of log_2_(Cy-3/Cy-5) values for internal control genes by using Excel (Office 2003; Microsoft Corp.) [24]. For each gene, the mean values were then calculated and a twofold difference was applied to select upregulated or downregulated genes in RA/OA FLSs or immature DC/bone marrow progenitors.

### Semiquantitative RT-PCR

To confirm the upregulation or downregulation of the selected gene (*MLN51*) on the microarray analysis and the expression of *MLN51 *after siRNA transfection, total RNAs were extracted from RA FLSs with Trizol reagent (Invitrogen) and purified with an RNeasy total RNA isolation kit (Qiagen) in accordance with the manufacturer's instructions. Total RNA (1 μg) was mixed with 50 μM oligo(dT)_20_, and 10 mM dNTP mixture, heated at 65°C for 5 minutes, and placed on ice for at least 1 minute. Then 10 × RT buffer (25 mM MgCl_2_, 0.1 M dithiothreitol, RNaseOUT™ (40 U)) and 1 μl of SuperScript™ III reverse transcriptase (200 U/μl; Invitrogen) were added, and the mixture was incubated at 42°C for 1 hour. The reaction was terminated by incubation at 75°C for 5 minutes followed by chilling on ice. The PCR was performed with the cDNA as template and certain gene specific-primers.

The following primers were used in this study: *hMLN51 *forward, 5'-AAGACACCGAGGACGAGGAATC-3', *hMLN51 *reverse, 5'-CCTTCCATAGCTTTCGCTGACG-3', product size 600 base pairs; *mMLN51 *forward, 5'-TCCCTGCCCTGCCCTGACTTTA-3', *mMLN51 *reverse, 5'-CCTCGCGTGCTGTGGGAACTCT-3', product size 800 bp; and glyceraldehyde-3-phosphate dehydrogenase (GAPDH) forward, 5'-CCACAGTCCATGCCATCAC-3', GAPDH reverse, 5'-TCCACCACCCTGTTGCTGTA-3', product size 500 bp. The initial cDNA content in each sample was normalized with the amount of GAPDH. Amplification reactions were performed in a 20 μl volume with 5 or 10 ng of each cDNA on a Perkin-Elmer DNA thermocycler 9600 Prism for 35 cycles. The PCR reactions were separated on 1.2% agarose gels and stained with ethidium bromide.

### Measurement of cytokine levels in rheumatoid arthritis synovial fluid

IL-1β and TNF-α were measured in the SFs with the Human Cytometric Bead Array (BD Pharmingen, San Diego), and GM-CSF was measured with the human ELISA kit (Endogen) in accordance with the manufacturer's instructions.

### Western blot analysis

RA FLS samples were lysed in boiled buffer containing 1% SDS. Each sample, containing a normalized amount of total protein (about 30 μg of protein), was separated by 10% SDS-PAGE and transferred to a nitrocellulose membrane. This was then immersed in blocking buffer (5% skimmed milk and 0.1% Tween 20 in PBS, pH 7.4) for 1 hour at room temperature and incubated with anti-hMLN51 rabbit serum (1:1,000 dilution) and anti-GAPDH (1:5,000 dilution) or anti-α-tubulin (1:5,000 dilution) in blocking buffer overnight at 4°C. Anti-hMLN51 serum was obtained from rabbits immunized with recombinant hMLN51 protein. After the incubation, the membrane was probed with horseradish peroxidase-labeled anti-rabbit IgG antibody (1:5,000 dilution) in PBS (containing of 0.05% Tween 20 and 5% skimmed milk powder) for 30 minutes at room temperature. The proteins in the membrane were detected by enhanced chemiluminescence (Amersham, Little Chalfont, Bucks., UK) and bands were detected by autoradiography with X-ray film (Fujifilm).

### Treatment of rheumatoid arthritis fibroblast-like synoviocytes with synovial fluid, cytokine or neutralizing antibodies

RA FLSs were cultured in 12-well plates in high-glucose DMEM supplemented with 10% FBS at 37°C in a 5% CO_2 _humidified incubator. For SF and cytokine treatments, RA FLSs were treated with SFs serially diluted in culture medium. Inflammatory cytokines (IL-1β and TNF-α; 100 ng/ml of each) and the growth factor (GM-CSF; 10 or 100 ng/ml) were obtained from PeproTech (Rocky Hill, NJ, USA) or BD Pharmingen (San Diego). Neutralizing monoclonal antibodies against GM-CSF (BVD2-23B6, IgG2a; 300 ng/ml), IL-1β (AS10, IgG1; 500 ng/ml) and TNF-α (MAb1, IgG1; 2 μg/ml) were purchased from BD Pharmingen (San Diego). RA FLSs were preincubated with these neutralizing antibodies for 1 hour. The trypan blue exclusion method was used for the evaluation of cell proliferation during all experiments.

### siRNA synthesis and transfection

siRNA synthesis was performed with the Silencer™ siRNA Cocktail Kit (RNase III; Ambion Inc., Austin, Texas, USA). The siRNA sequence was used for targeted silencing of human *MLN51 *(GenBank accession number NM007359) and mouse *MLN51 *(GenBank accession number AJ292072). The oligonucleotides used for the dsRNA synthesis were, in *hMLN51*, 5'-TAATACGACTCACTATAGGGTACTCGTAAGATGGCGGACCGG-3' and 5'-TAATACGACTCACTATAGGGTCCGTCCCCACTTTGCCTC-3', and in *mMLN51*, 5'-TAATACGACTCACTATAGGGTACTCGTAAGATGGCGGACCGG-3' and 5'-TAATACGACTCA CTATAGGGTACTCTGCCTCTCCCCAGTCAC-3'. The siRNA sequences were selected in size ranging from 228 to 686 bp, as described previously [25,26]. The siRNA synthesis was performed in accordance with the manufacturer's protocol. Non-silencing or negative control siRNA (Silencer Negative Control no. 2 siRNA; Ambion Inc.) is an irrelevant siRNA with random nucleotides and no known specificity. RA FLSs (RA 2–14, at passage 5; 10^4 ^per well) and BC-1 cells (10^4 ^per well) were seeded in 24-well plates in DMEM supplemented with 10% FBS and Iscove's modified Dulbecco's medium (containing 10% FCS), respectively. The cells were transfected with the siRNA (4 μg) on the next day, with the GenePORTER 2 Transfection reagent™ (Gene Therapy Systems, San Diego, CA, USA) in accordance with the manufacturer's protocol. At 24 hours after transfection, fresh culture medium was added to the medium. Cells were harvested every day and counted. Total RNA extracted from the transfected cells was used to perform semiquantitative RT-PCR.

### Statistical analysis

The results are expressed as means ± SD. The Mann – Whitney *U *test was used for all statistical analysis. *p *< 0.05 was considered significant.

## Results and discussion

RA is a heterogeneous autoimmune disease. However, these heterogeneous chronic diseases were recently able to be monitored in line with their gene expression patterns by microarray-based molecular studies [27]. The histology of RA affected joints indicates chronic inflammation with hyperplasia in the synovial lining cells. It is now well established that FLSs actively participate in RA synovitis and that FLSs in RA joints aggressively proliferate to form a pannus, eventually destroying articular bone and cartilage [28,29]. Several cytokines, such as IL-1β, TNF-α and IL-6, have been described in association with the proliferative response of FLSs. In trials of these therapeutic agents, however, responses were not achieved in a significant proportion of the patients, suggesting that some important factor(s) still remain to be discovered.

To our knowledge this report is the first demonstration that the *MLN51 *is essential for the hyperproliferation of RA FLSs in line with GM-CSF signaling in RA pathogenesis. Our results show that the SF-mediated growth of RA FLSs was markedly blocked by anti-GM-CSF neutralizing antibody, and additionally that growth-retarded RAFLSs recovered their proliferative capacity by the addition of GM-CSF. These results indicate that GM-CSF in SF is important in the hyperproliferation of RA FLSs. In contrast, in the microarray analysis, semiquantitative RT-PCR and Western blot analysis experiments, we found that the *MLN51 *was consistently overexpressed in the hyperactive RA FLSs at low passages or the RA FLSs cultured in the presence of SF. *MLN51 *knock-down by siRNA completely blocked the GM-CSF/SF-mediated proliferation capacity of RA FLSs, suggesting that the *MLN51 *gene is strongly involved in the pathogenesis of RA.

**Figure 1:**
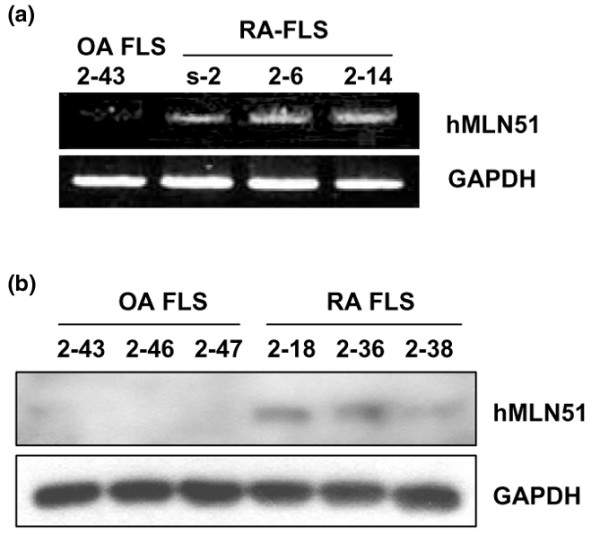
*MLN51 *expression is upregulated in rheumatoid arthritis FLSs compared with osteoarthritis FLSs. **(a) **Total RNA sample (1 μg) was extracted from three rheumatoid arthritis (RA) fibroblast-like synoviocytes (FLSs) and one osteoarthritis (OA) FLS with Trizol reagent. RT-PCR was performed with 5 ng of cDNA as a template and *MLN51*-specific or GAPDH-specific primers. The band for OA FLSs resulted from one (2–43) of the three OA FLSs. **(b) **Western blot analysis of MLN51 in FLS samples. RA FLSs (2–18, 2–36 and 2–38) and OA FLSs (2–43, 2–46 and 2–47) isolated from each patient were seeded at 5 × 10^4 ^cells per well in a six-well plate. FLSs grown in high-glucose DMEM supplemented with 10% FBS were harvested, separated by 10% SDS-PAGE, transferred to a nitrocellulose membrane and then proved with anti-hMLN51 rabbit serum (1:1,000 dilution) and horseradish peroxidase-conjugated anti-rabbit IgG (1:5,000 dilution). Data in (a) and (b) are representative of three or four separate experiments.

We extracted total RNA from RA FLSs and OA FLSs, which were labeled by cDNA synthesis and ultimately hybridized to a HI380 microarray containing 384 cDNA clones. The differential hybridization was performed with Cy-5-labeled RA cDNA and Cy-3-labeled OA cDNA probes. Through the microarray analysis, we found that *MLN51*, a novel gene in association with RA, was markedly upregulated among the many upregulated genes selected on the basis of their immunologic characteristics (Table 1). *MLN51 *overexpression in RA FLSs was confirmed by RT-PCR analysis with three different RA FLS samples (Figure 1a) and by Western blot experiments with additional three different RA FLS samples (Figure 1b).

**Figure 2:**
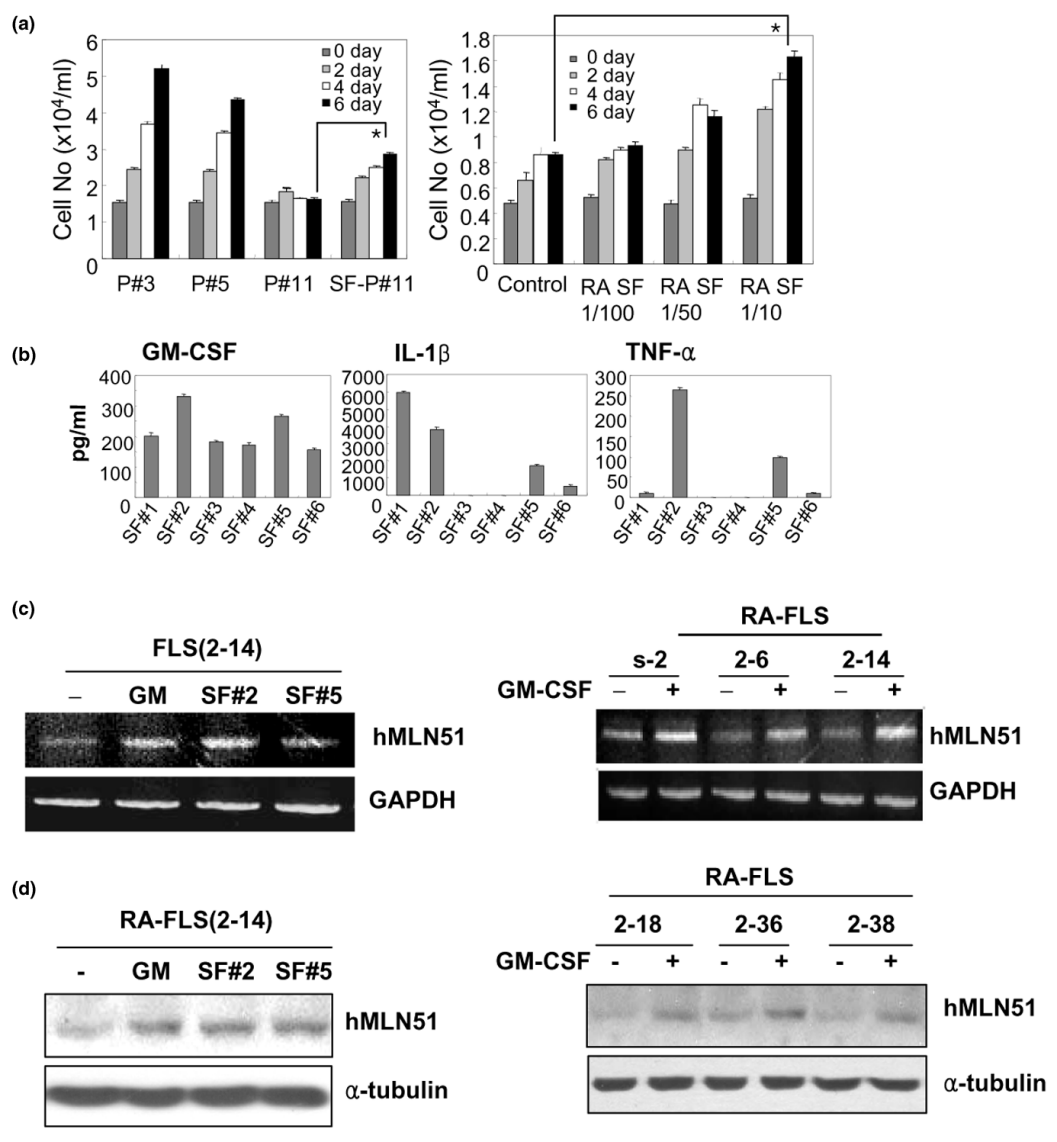
The growth kinetics of RA FLSs at different passages or in SF-treated cultures. **(a) **Rheumatoid arthritis (RA) fibroblast-like synoviocytes (FLSs) at passages 3 (P#3), 5 (P#5) and 11 (P#11) were used to measure their growth kinetics. RA FLSs (2–14) at passage 11 was treated with diluted synovial fluid (SF) no. 2 (1/100, 1/50 or 1/10 dilutions) to evaluate growth recovery; the culture was incubated for 6 days and the concentrations of treated SF were as follows: left panel, 1/10 dilution; right panel, 1/100, 1/50 and 1/10 dilutions of SF. *p < 0.01. **(b) **The concentration of granulocyte – macrophage colony-stimulating factor (GM-CSF) and cytokines in SFs from each patient with RA. Six SF samples were analyzed for their concentrations of GM-CSF and other cytokines with an ELISA kit and a Cytometric Bead Array kit. The results in (a) and (b) are means ± SD obtained from single experiments performed in triplicate cultures. **(c, d) ***MLN51 *expression was quantified by RT-PCR (c) and Western blot analysis (d) in SF-treated or GM-CSF-treated RA FLSs. The high-passage-number RA FLSs (RA 2–14, passage 11) were treated with GM-CSF (100 ng/ml) or 1/10-diluted SF every 2 days for 6 days (left panels). In addition, the expression of *MLN51 *was evaluated in six different RA FLS samples treated with or without GM-CSF (100 ng/ml; right panels). See the Materials and methods section for a detailed description. Data in (c) and (d) are representative of three separate experiments.

**Figure 3:**
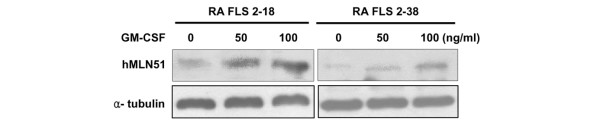
Western blot analysis of hMLN51 in RA fibroblast-like synoviocytes (FLSs) treated with GM-CSF. Rheumatoid arthritis (RA) FLSs (2–18 and 2–38) isolated from the two patients with RA were seeded at 5 × 10^4 ^cells per well in a six-well plate. FLSs grown in high-glucose DMEM supplemented with 10% FBS were cultured further in the presence of granulocyte – macrophage colony-stimulating factor (GM-CSF) at 0, 50 and 100 ng/ml for 6 hours. Cells were harvested, separated by 10% SDS-PAGE, transferred to a nitrocellulose membrane and then proved with anti-hMLN51 rabbit serum (1:1,000 dilution) and horseradish peroxidase-conjugated anti-rabbit IgG (1:5,000 dilution). This result is representative of three separate experiments.

We next investigated whether SFs have an effect on the growth rate of RA FLSs, what kinds of factors in the SFs are involved in the proliferation of RA FLSs, and whether the factors have a role in the expression of *MLN51*. We determined the growth kinetics of FLSs at different passages and SF-treated RA FLSs. The RA FLSs at passage 11 showed obvious growth retardation (Figure 2a, left panel); however, the same sample clearly recovered from its growth retardation when cultured in the presence of 10-fold-diluted SF (Figure 2a, right panel). We next quantified inflammatory cytokine levels in SFs (Figure 2b). The results indicated that GM-CSF in all SFs from six patients with RA exists at nearly equal levels, in contrast with other inflammatory cytokines (such as IL-1β and TNF-α). We found that *MLN51 *expression in RA FLSs was upregulated in mRNA level (Figure 2c, left panel) and protein level (Figure 2d, left panel) by treatment of cultures not only with SFs but also with GM-CSF. The upregulation of *MLN51 *by GM-CSF treatment was also confirmed in six different RA FLS samples by RT-PCR (s-2, 2–6 and 2–14; Figure 2c, right panel) and by Western blot analysis (2–18, 2–36, 2–38; Figure 2d, right panel). Moreover, in both RA FLS samples (2–18 and 2–38), the MLN51 protein expression was enhanced by GM-CSF treatment in a dose-dependent manner (Figure 3). These results strongly suggest that the growth rate recovery of RA FLSs by SF or GM-CSF is associated with the expression of *MLN51*.

**Figure 4:**
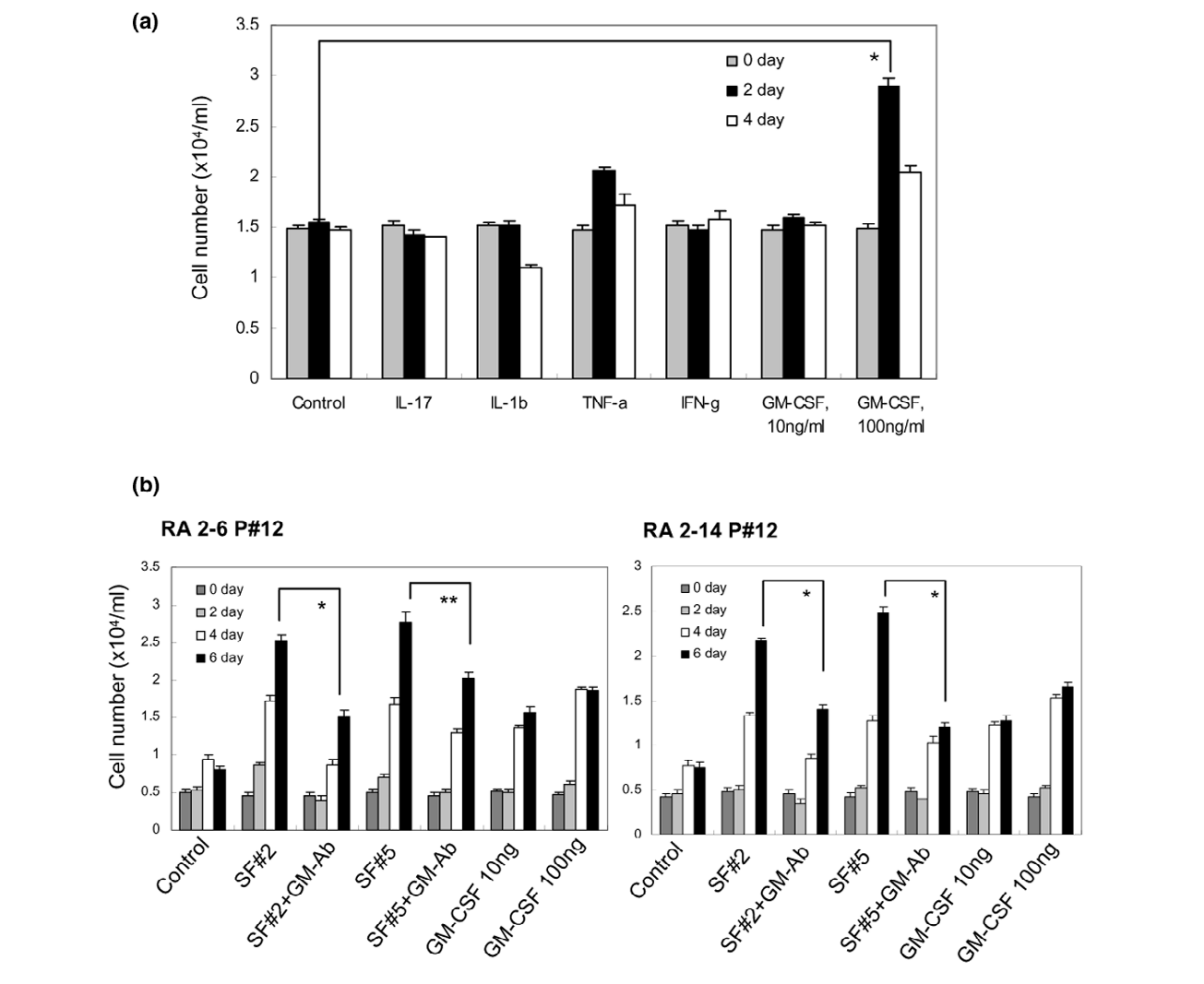
Effects of GM-CSF and cytokines on the growth of high-passage-number RA FLSs. **(a) **Rheumatoid arthritis (RA) fibroblast-like synoviocytes (FLSs) 2–14 (at passage 11) were seeded at 1.5 × 10^4 ^cells per well in triplicate in a 24-well plate. Cells cultured in high-glucose DMEM supplemented with 10% FBS were treated with cytokines (each at 100 ng/ml) or 10 or 100 ng/ml granulocyte – macrophage colony-stimulating factor (GM-CSF) on day 0. Cells were harvested every 2 days and counted. **(b) **The growth restoration of RA FLSs mediated by synovial fluid (SF) was markedly inhibited by neutralizing antibody against GM-CSF. RA FLSs (2–6 and 2–14) at passage 12 were cultured at 5 × 10^3 ^cells per well in a 24-well plate. FLSs in culture were treated with GM-CSF (10 or 100 ng/ml) or SF at 1/10 dilution every 2 days for 6 days. SF-treated FLSs were cultured in the presence or absence of anti-GM-CSF neutralizing antibody (300 ng/ml). Cells were counted and assessed for viability by trypan blue staining every 2 days. The results are means ± SD obtained from single experiments performed in triplicate cultures. *p < 0.01; **p < 0.05.

There are many kinds of different cytokines and growth factors in the RA joint microenvironments. To identify factors having an effect on the growth of RA FLSs, we investigated the inflammatory cytokines and growth factors on the growth of RA FLSs (2–14) *in vitro*. The results indicated that GM-CSF and TNF-α may have an effect on the growth rate recovery of the high-passage-number RA FLSs. GM-CSF and TNF-α treatment resulted on approximately 2.0-fold and 1.3-fold increases in the proliferation of RA FLSs, respectively, compared with that of untreated controls (Figure 4a). These results support the notion that resident joint cells (chondrocytes and synovial fibroblasts) produce GM-CSF in culture in response to TNF-α and IL-1β [30,31]. However, our result showed that IL-1β did not induce active proliferation of RA FLSs, indicating that IL-1β may not be a key factor in active RA FLS proliferation. In contrast, the growth rate recovery of the high-passage-number RA FLSs was achieved *in vitro *when the cells were treated with 100 ng/ml GM-CSF (a significant difference from the control cells), although GM-CSF concentrations measured in SFs of patients with RA were a maximum of 400 pg/ml and SF treatment induced active proliferation of RA FLSs. This suggested that the combinations of various proinflammatory cytokines or other factors together with GM-CSF in the SF may be involved in RA pathogenesis *in vivo*. To address the effects of GM-CSF in SF on the growth of RA FLSs, we cultured the RA FLSs in culture media containing SF and anti-GM-CSF mAb or a recombinant GM-CSF. Incubation of the two different RA FLSs with SFs containing anti-GM-CSF mAb significantly impaired the SF-mediated proliferation efficacy of FLSs (Figure 4b). These results suggest that the GM-CSF in SF has a key role in the hyperproliferation of RA FLSs, further supporting the results above. The cell viability for all cultures (data not shown) was 98 to 99%. The recovery of the growth-retarded RA FLS proliferation capacity was obviously improved by GM-CSF in a dose-dependent manner.

**Figure 5:**
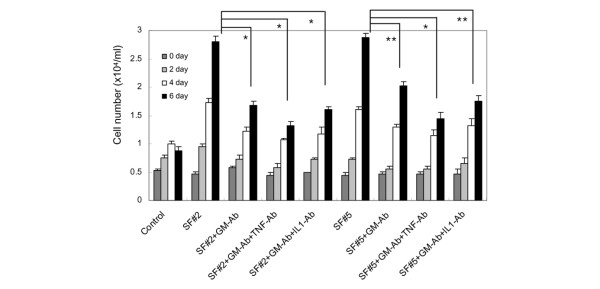
Inhibitory effects of neutralizing antibodies to cytokines on the SF-mediated proliferation capacity of RA FLSs. Rheumatoid arthritis (RA) fibroblast-like synoviocytes (FLSs; 2–14) at passage 12, at a concentration of 5 × 10^3 ^cells per well in a 24-well plate, were cultured in the presence of synovial fluid (SF) at a dilution of 1/10 or in the presence of SF together with anti-granulocyte – macrophage colony-stimulating factor (300 ng/ml), anti-IL-1β (500 ng/ml) or anti-TNF-α (2 μg/ml) neutralizing antibodies. RA FLSs were preincubated for 1 hour with these neutralizing antibodies. Two different SFs were added every 2 days in the presence or absence of neutralizing monoclonal antibody. Cells were harvested every 2 days and their viability was assessed by trypan blue staining. The results are means ± SD obtained from single experiments performed in triplicate cultures. *p < 0.01; **p < 0.05.

We cultured RA FLSs (2–14) in SF-containing medium in the presence of anti-GM-CSF, anti-GM-CSF plus anti-IL-1β or anti-GM-CSF plus anti-TNF-α mAbs to investigate the effects of IL-1β or TNF-α in SF on recovery of the growth of RA FLSs. As shown in Figure 5, cultures treated with both anti-GM-CSF and anti-TNF-α mAbs showed slightly more suppression than the cultures treated with anti-GM-CSF mAb alone, in terms of SF-mediated FLS proliferation. Our results in Figures 4b and 5 suggest that not only GM-CSF, but also some other proinflammatory cytokines such as TNF-α, are likely to be involved in the hyperproliferation of RA FLSs. However, anti-IL-1β mAb did not have a significant effect on the SF-mediated proliferation of RA FLSs. These cytokine effects on the FLS proliferation were similar to those on the *MLN51 *gene expression level (data not shown).

**Figure 6:**
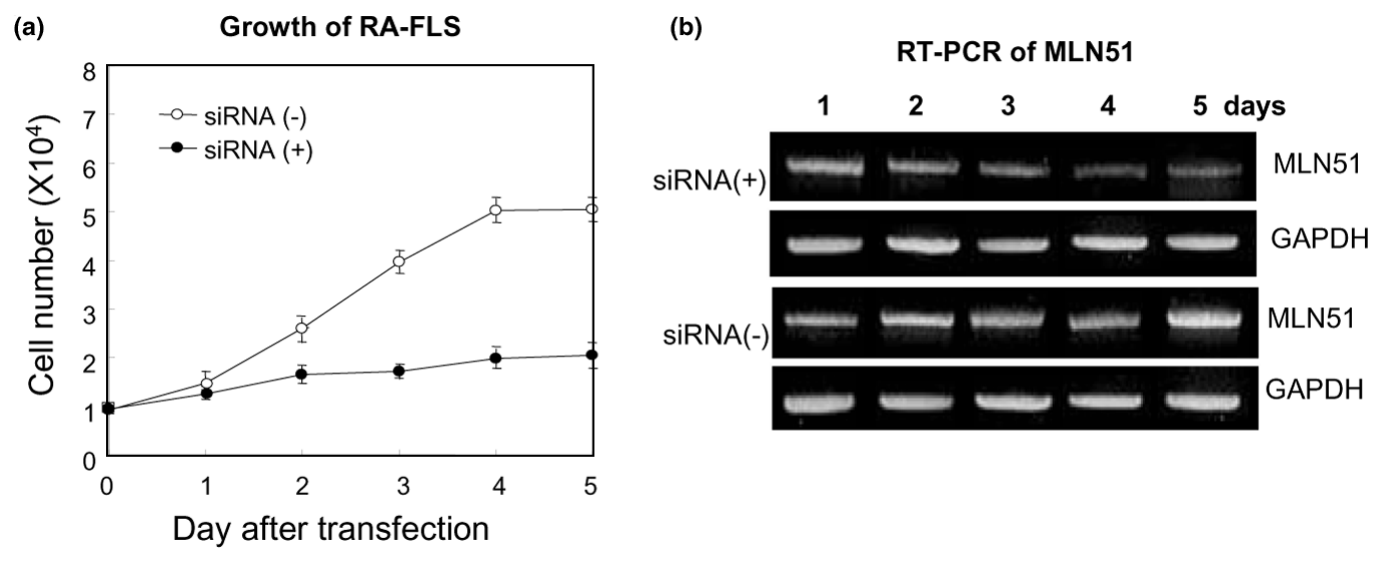
Effects of *MLN51*-knock-down on the growth of RA FLSs. **(a) **Fibroblast-like synoviocytes (FLSs; 10^4 ^cells; rheumatoid arthritis (RA) 2–14, at passage 5) were transfected with 4 μg of *hMLN51 *siRNA or control siRNA. The transfected cells were cultured in growth medium and counted each day for 5 days. The trypan blue exclusion method was used to evaluate cell proliferation. The results are means ± SD from single experiments performed in triplicate cultures. **(b) ***MLN51 *expression according to the above result was monitored by RT-PCR. See the Materials and methods section for a detailed description. This result is representative of two separate experiments.

To examine a specific requirement for the *MLN51 *gene in cell proliferation, siRNA prepared from the 5' region of human *MLN51 *cDNA was introduced into passage 5 of RA FLSs (2–14). The growth kinetics of the transfected RA FLSs was monitored for 5 days (Figure 6a) and the level of the corresponding *MLN51 *mRNA was measured by semiquantitative RT-PCR (Figure 6b). As shown in Figure 6, treatment of the FLSs with *hMLN51*-siRNA caused complete abrogation of RA FLS proliferation, whereas treatment with control siRNA was without effect. These results strongly suggest that the *MLN51 *gene has a crucial role in the hyperproliferation of RA FLSs.

**Figure 7:**
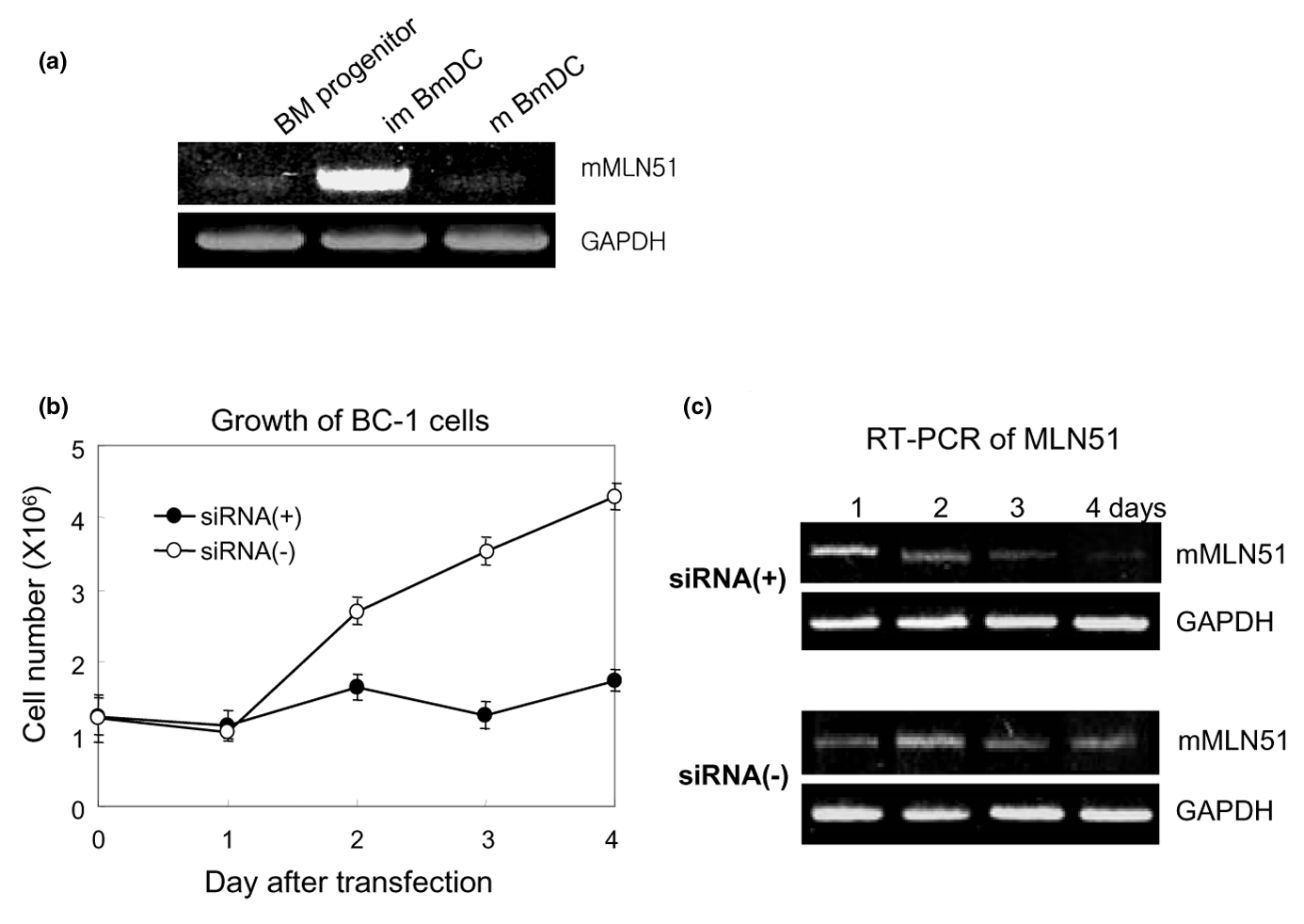
*MLN51 *expression in dendritic cells (DCs) and its effect on the proliferation of immature DCs. **(a) ***MLN51 *expression is upregulated only in immature bone marrow-derived dendritic cells (BmDCs) in contrast with bone marrow progenitor cells or mature BmDCs. Total RNA was extracted from each sample with Trizol reagent. RT-PCR was performed with mouse *MLN51 *primers. **(b) **BC-1 cells (10^6^) were transfected with 4 μg of *mMLN51 *siRNA (siRNA(+)) or control siRNA (siRNA(-)). The transfected cells were harvested every day and assessed for their proliferation over a period of 4 days. The results are means ± SD obtained from single experiments performed in triplicate cultures. **(c) **Total RNA was extracted each day from the transfected BC-1 cell cultures. mMLN51 was assessed in each sample by RT-PCR as described previously with primers. Results in (a) and (c) are representative of three separate experiments.

We next generated the BmDCs from the DBA/1J mouse, which is a frequently used animal model for arthritis. It is known that the DBA/1 mouse strain has a H-2^q ^haplotype and readily develops arthritis after immunization with heterologous or autologous type II collagen of rat, bovine or chick CII origin [32]. In addition, DCs are particularly relevant in the pathogenesis of most inflammatory arthropathies because of their potent antigen-presenting capacity and their unique ability to activate naïve T cells [33-35]. In addition, DC populations have been described in line with synovitis in RA, although a functional contribution to the disease remains difficult to assess [36-38]. The immature BmDCs were generated from bone marrow progenitors by culturing the progenitors in the presence of GM-CSF alone. Immature BmDCs were matured with lipopolysaccharide and anti-CD40. We then performed semiquantitative RT-PCR of mouse *MLN51 *gene expression with the aim of confirming the differences observed in cDNA microarray analysis. As shown in Figure 7a, the *MLN51 *gene was highly expressed only in the immature BmDCs and barely detected in the bone marrow progenitor or mature BmDCs. These results suggest that the expression of *MLN51 *is associated with the GM-CSF treatment. We hypothesized that the *MLN51 *gene might have one or more important roles in immature DCs in line with their specific biological functions or with some aspects of cell viability. We investigated a function of *MLN51 *on the growth of DCs by using BC-1 cells (an immature DC cell line). BC-1 cells transfected with *MLN51 *siRNA were harvested daily, and cell proliferation and *MLN51 *mRNA expression were measured by RT-PCR. As shown in RA-FLSs (Figure 6), the transfection of *MLN51*-specific siRNA abrogated the proliferation of BC-1 cells (Figure 7b) resulting from the *MLN51 *knock-down (Figure 7c). These results indicate that the *MLN51 *gene, identified in breast cancers, is important in the proliferation of not only FLSs but also established DC cell lines.

In summary, our results strongly suggest that the *MLN51 *gene, whose expression depends upon GM-CSF signaling, may have a crucial role in the hyperproliferation of FLSs in the pathogenesis of RA.

## Conclusion

We have identified and demonstrated for the first time that the *MLN51 *is highly expressed in RA FLSs. *MLN51 *overexpression in the RA FLSs is associated with GM-CSF in the SF of patients with RA. The *MLN51 *seems to have a critical role in the hyperproliferation of FLSs in RA pathogenesis. *MLN51 *could be an attractive target for the development of new RA therapeutics.

## Abbreviations

BmDC = bone marrow-derived dendritic cell; bp, base pairs; DC = dendritic cell; DMEM = Dulbecco's modified Eagle's medium; FCS = fetal calf serum; FLS = fibroblast-like synoviocyte; GM-CSF = granulocyte – macrophage colony-stimulating factor; IL = interleukin; mAb = monoclonal antibody; *MLN51 *= metastatic lymph node 51; OA = osteoarthritis; RA = rheumatoid arthritis; SF = synovial fluid; siRNA = small interfering RNA; TNF = tumor necrosis factor.

## Competing interests

The authors applied for a patent relating to this manuscript. However, no reimbursement or financial support was provided by the institute with regard to the patent application.

## Authors' contributions

YSB was responsible for most of the data analysis as well as drafting the manuscript. DSL, together with YSB, was responsible for study design coordination and the writing of this manuscript, and also for interpretation and discussion of the data. JJ, YEC and YJ performed most of the studies and were responsible for the execution of most of the experiments. SAY and WUK provided the patients' FLS and SF samples, liaised with the St Mary Hospital and gave us valuable assistance during the period of experimentation and manuscript preparation. All authors read and approved the final manuscript.

## Figures and Tables

**Table 1:** List of genes upregulated in rheumatoid arthritis/osteoarthritis microarray analysis (HI380)

Genes upregulated in RA fibroblast-like synoviocytes^a^	Functions	RA/OA FLS ratio^b^
CD1A antigen, α polypeptide	T cell activation	2.6
CD3D antigen, δ polypeptide (TiT3 complex)	T cell activation	3.7
*MLN51 *gene	Highly expressed in immature DCs	6.8
CD6 antigen	T cell activation	2.5
CD21 antigen	B cell activation	2.1
CD24 antigen (small cell lung carcinoma cluster 4 antigen)	Unknown for human CD24	4.3
CD26 antigen	T cell activation	4.1
CD32 antigen (Fcγ receptor II)	Formation of immune complexes	2.2
CD33 antigen (gp67)	Inhibition of myeloid cell proliferation	3.8
CD36 antigen (collagen type I receptor, thrombospondin receptor)	Recognition and phagocytosis of apoptotic cells	2.5
CD42b antigen	Adhesion or migration	4.5
CD79A antigen (immunoglobulin-associated α)	B cell activation	2.4
CD171 antigen	T cell activation	3.6
CD206 antigen (macrophage mannose receptor)	Apoptosis or endocytosis	2.8
B cell linker protein (SLP65: BLNK)	B cell activation	4.0
Ig superfamily protein (Z39IG)	Formation of immune complexes	4.2
T cell transcription factor 4 (TCF-4)	Enhancement of the release of extracellular matrix proteins	2.1
IL-1 receptor type I	T cell activation	2.4
IL-7 receptor	T and B cell activation	2.5
IL-8	Migration	3.0
IL-9	T and mast cell activation	2.7
IL-17	T cell activation	4.6
Colony-stimulating factor 2 receptor, β, low-affinity (granulocyte – macrophage); CSF-2RB	Cell cycle regulation	2.3
Colony-stimulating factor 3 receptor (granulocyte)	Cell cycle regulation	2.9
Methyl-CpG-binding domain protein 1 (MBD1)	Cell cycle regulation	2.2

DCs, dendritic cells; FLS, fibroblast-like synoviocyte; OA, osteoarthritis; RA, rheumatoid arthritis. ^a^RA FLS 2–14 was used for this experiment. Values were obtained from one (OA 2–43) of three OA FLSs. ^b^This result was based on single experiments performed in triplicates, being similar to the cases performed with additional FLSs.
